# Impairment of insulin signalling in peripheral tissue fails to extend murine lifespan

**DOI:** 10.1111/acel.12610

**Published:** 2017-05-22

**Authors:** Troy L. Merry, Doreen Kuhlow, Beate Laube, Doris Pöhlmann, Andreas F. H. Pfeiffer, C. Ronald Kahn, Michael Ristow, Kim Zarse

**Affiliations:** ^1^ Energy Metabolism Laboratory Swiss Federal Institute of Technology (ETH) Zurich‐Schwerzenbach Schwerzenbach, Zurich Switzerland; ^2^ School of Medical Sciences University of Auckland Auckland New Zealand; ^3^ Department of Human Nutrition Friedrich Schiller‐University Jena Germany; ^4^ Department of Human Nutrition German Institute of Human Nutrition Potsdam‐Rehbrücke Nuthetal Germany; ^5^ Medizinische Klinik für Endokrinologie Diabetes und Ernährungsmedizin Charité University Medicine Berlin Berlin Germany; ^6^ Section on Integrative Physiology and Metabolism Joslin Diabetes Center and Department of Medicine Boston MA USA

**Keywords:** aging, fat mass, glucose tolerance, healthspan, insulin sensitivity, lifespan, longevity

## Abstract

Impaired insulin/IGF1 signalling has been shown to extend lifespan in model organisms ranging from yeast to mammals. Here we sought to determine the effect of targeted disruption of the insulin receptor (IR) in non‐neuronal tissues of adult mice on the lifespan. We induced hemizygous (PerIRKO ^+/−^) or homozygous (PerIRKO ^−/−^) disruption of the IR in peripheral tissue of 15‐weeks‐old mice using a tamoxifen‐inducible Cre transgenic mouse with only peripheral tissue expression, and subsequently monitored glucose metabolism, insulin signalling and spontaneous death rates over 4 years. Complete peripheral IR disruption resulted in a diabetic phenotype with increased blood glucose and plasma insulin levels in young mice. Although blood glucose levels returned to normal, and fat mass was reduced in aged PerIRKO ^−/−^ mice, their lifespan was reduced. By contrast, heterozygous disruption had no effect on lifespan. This was despite young male PerIRKO ^+/−^ mice showing reduced fat mass and mild increase in hepatic insulin sensitivity. In conflict with findings in metazoans like *Caenorhabditis elegans* and *Drosophila melanogaster*, our results suggest that heterozygous impairment of the insulin signalling limited to peripheral tissues of adult mice fails to extend lifespan despite increased systemic insulin sensitivity, while homozygous impairment shortens lifespan.

## Introduction

Longevity is determined by a complex interaction between environmental and genetic factors (Martin *et al*., 1996). One of the most successful interventions to delay the onset of aging‐associated diseases and increase life expectancy is the chronic reduction in food intake, that is calorie restriction (CR) (Weindruch & Walford, 1988). One of the mechanisms through which CR may extend lifespan is through reduced activation of insulin/IGF1 signalling (Friedman & Johnson, 1988; Kenyon *et al*., 1993; Brown‐Borg *et al*., 1996; Morris *et al*., 1996; Clancy *et al*., 2001; Tatar *et al*., 2001, 2003; Blüher *et al*., 2003; Holzenberger *et al*., 2003; Selman *et al*., 2008; Nojima *et al*., 2013).

Following food intake, insulin is released and acts via the insulin receptor (IR) to facilitate the metabolism of glucose (Saltiel & Kahn, 2001). Altering glucose metabolism via the inhibition of glycolysis or the impairment of insulin/IGF1 signalling consistently extends lifespan in *C. elegans* and *D. melanogaster* (Friedman & Johnson, 1988; Kenyon *et al*., 1993; Morris *et al*., 1996; Clancy *et al*., 2001; Tatar *et al*., 2001). However, the effect of insulin/IGF1 signalling cascade inhibition on longevity in mammals is less clear. In mammals, prenatal complete ablation of the insulin receptor (IR) (Accili *et al*., 1996) or IGF1 receptor (IGF1R) globally (Liu *et al*., 1993; Holzenberger *et al*., 2003) shortens lifespan, whereas ablation of the IR in some tissues such as the liver and pancreas induces a diabetic phenotype (Kulkarni *et al*., 1999; Michael *et al*., 2000). The IR appears to play a central role in normal development (Accili *et al*., 1996; Okamoto *et al*., 2004), and central nervous system (CNS) IR expression in adulthood is required for the maintenance of glucose homeostasis (Bruning *et al*., 2000; Koch *et al*., 2008). This suggests that the absence of the IR in peripheral tissues during early development, and in the CNS during adulthood may contribute to reduced lifespan and diabetic phenotype seen in some prenatal IR knockout models.

Interestingly, aP2‐Cre‐mediated deletion of the IR, which primarily targets adipose tissue but may also act on several other nervous system and peripheral tissues (Pomplun *et al*., 2007; Martens *et al*., 2010), as well as the disruption of the insulin/IGF1 signalling pathway downstream of the IR (Taguchi *et al*., 2007; Selman *et al*., 2008; Nojima *et al*., 2013), is effective in prolonging lifespan in rodents. Furthermore, some genetic variations in the insulin/IGF1 pathway are associated with increased human lifespan expectancy (Suh *et al*., 2008; Pawlikowska *et al*., 2009). This is consistent with the requirement of the IR to facilitate metabolism of glucose in peripheral tissue and indicates that some degree of IR signalling is probably required during development and adulthood for normal life expectancy.

In all of these studies, however, the disruptions in insulin signalling were created using systems that were active from early developmental stages, so little is known about the effects of altered insulin signalling on longevity when the alteration is limited to adulthood. We have previously shown that partial disruption of the IR in mammalian cells causes adaptations, and similar adaptations extend the lifespan of *C. elegans* (Zarse *et al*., 2012). In the present study, we determined the effect of partial or complete adult‐induced peripheral tissue IR disruption on metabolism and lifespan of mice. We report that homozygous adult‐induced peripheral tissue IR disruption leads to a diabetic phenotype which reduced lifespan, while hemizygous peripheral tissue IR disruption did not affect lifespan despite male mice showing evidence of enhanced insulin sensitivity.

## Results

### Insulin receptor (IR) disruption

We firstly assessed the effectiveness of the oestrogen receptor (ER) antagonist, tamoxifen, to initiate Cre‐ER^T2^‐induced recombination and disrupt the expression of the IR in peripheral (Per) tissues (liver, white adipose tissue and skeletal muscle) of PerIRKO^−/−^ and PerIRKO^−/+^ mice, respectively. Three weeks following tamoxifen (TX) treatment, IR expression in these tissues was completely ablated in PerIRKO^−/−^, and partially reduced in PerIRKO^−/+^ adult mice (Fig. S1A, Supporting information). Brown adipose IR was partially ablated in both PerIRKO^−/−^ and PerIRKO^−/+^ (Fig. S1A, Supporting information). Consistent with previous observations that tamoxifen does not effectively activate the Cre recombinase in the CNS (Koch *et al*., 2008), PerIRKO^−/−^ and PerIRKO^−/+^ whole‐brain, hypothalamus and pituitary gland IR expression was unaltered (Fig. S1, Supporting information).

### Peripheral tissue IR disruption in adult mice impairs blood glucose regulation

The effect of complete disruption of the IR in peripheral tissue of adult mice (PerIRKO^−/−^) on NMRI‐based body composition and blood glucose levels was assessed 4–6 weeks following TX or vehicle (Veh) treatment. Body mass and composition of male PerIRKO^−/−^ mice were not different from control mice (Fig. S2A–C, Supporting information). Female PerIRKO^−/−^ mice treated with TX, but not Veh, showed reduced body mass compared with control mice; however, relative body composition was not effected (Fig. S2D–F, Supporting information). While male and female Veh‐ and TX‐treated PerIRKO^−/−^ mice had similar fasted blood glucose to that of control, TX‐treated PerIRKO^−/−^ mice showed elevated fed blood glucose (Fig. S2G,H, Supporting information). Consistent with this, both male and female TX‐treated PerIRKO^−/−^ mice showed greatly increased fed plasma insulin levels, while fasted insulin levels were only increased in female PerIRKO^−/−^ mice (Fig. S2I,J, Supporting information). This indicates that the complete disruption of the IR in peripheral tissue of adult mice impairs glucose regulation and insulin sensitivity. In addition, plasma adiponectin was increased in both male and female PerIRKO^−/−^ mice, whereas plasma leptin was only increased in females, and plasma IL‐6 tended to be elevated in males (Fig. S2K–M, Supporting information). These metabolic characteristics are largely consistent with the previously described phenotype of PerIRKO^−/−^ mice (Koch *et al*., 2008), and therefore, detailed analysis of the metabolic phenotype was continued for the previously unreported PerIRKO^−/+^ mice only.

### Reduced peripheral tissue IR expression alters male mice body composition

**Figure 1:**
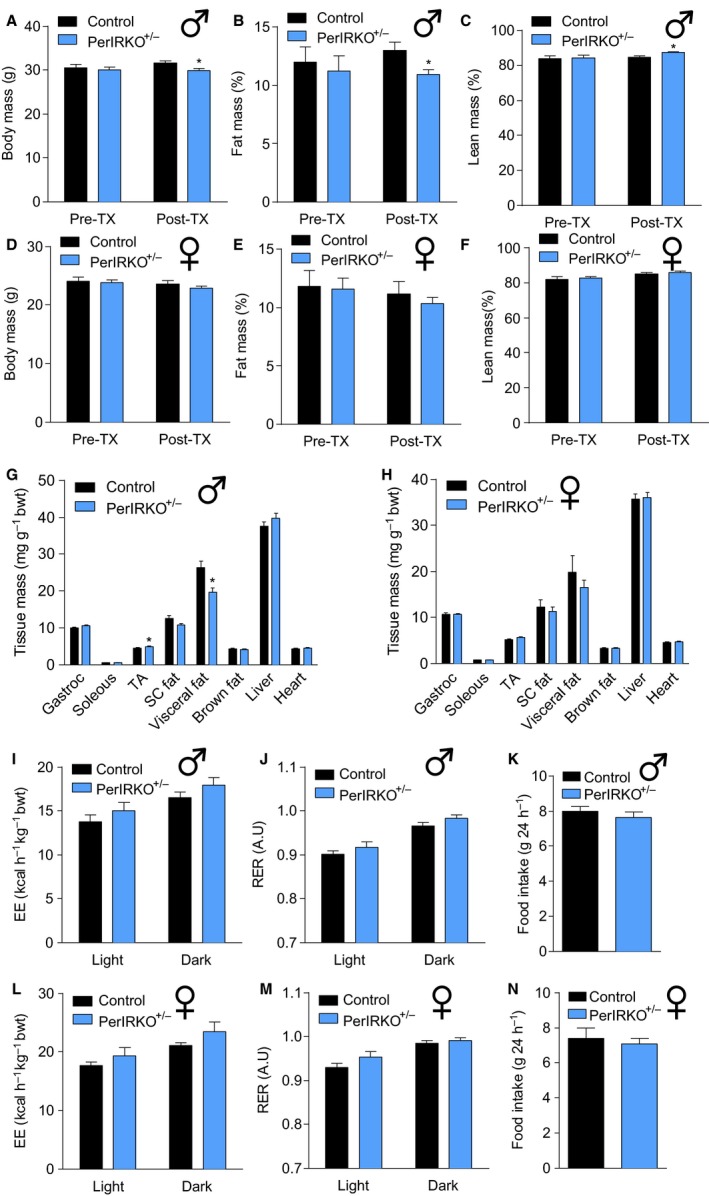
The effect of partial peripheral tissue IR disruption on body composition and energy expenditure in adult mice. Four to six weeks following tamoxifen (TX) treatment, male (A–C) and female (D–F) control and PerIRKO ^−/+^ mice body mass and body composition (NMRI) were measured before being sacrificed and the mass of male (G) and female (H) tissue was determined. Male (I–K) and female (L–N) energy expenditure, respiratory exchange ratio (RER) and food intake were measured during light and dark cycles for 48 h. Results are shown as means ± SE for *n* = 12–13 per group for body composition and tissue mass, and *n* = 9–10 per group for energy expenditure and other parameters. Significance was determined using two‐tailed Student's *t*‐test vs. control; *P < 0.05. bwt, body weight; Gastroc, *gastrocnemius* muscle; TA,* tibialis* anterior muscle; SC, subcutaneous.

To assess whether partial disruption of the IR in peripheral tissue affects body composition, body mass, fat mass or lean mass of control and PerIRKO^−/+^ mice, those parameters were determined by NMRI prior to and 4 weeks following TX treatment (Fig. 1A–F). Reducing peripheral tissue IR expression (PerIRKO^−/+^) resulted in lower body mass, reduced fat mass and increased percentage of lean mass in male mice (Fig. 1A–C). This is consistent with male PerIRKO^−/+^ having increased tibialis anterior (TA) muscle and reduced visceral fat pad mass relative to body weight (Fig. 1G). However, this modest reduction in relative fat mass was not associated with a detectable increase in whole‐body energy expenditure, altered metabolic substrate preference (RER) or reduction in food intake (Fig. 1I–K). In female PerIRKO^−/+^, there were no differences from control in body mass, body composition (Fig. 1D–F,H), whole‐body metabolism and food intake (Fig 1. L–N).

### Reduced peripheral tissue IR expression does not affect glucose homeostasis, but increases insulin sensitivity in male mice

**Figure 2:**
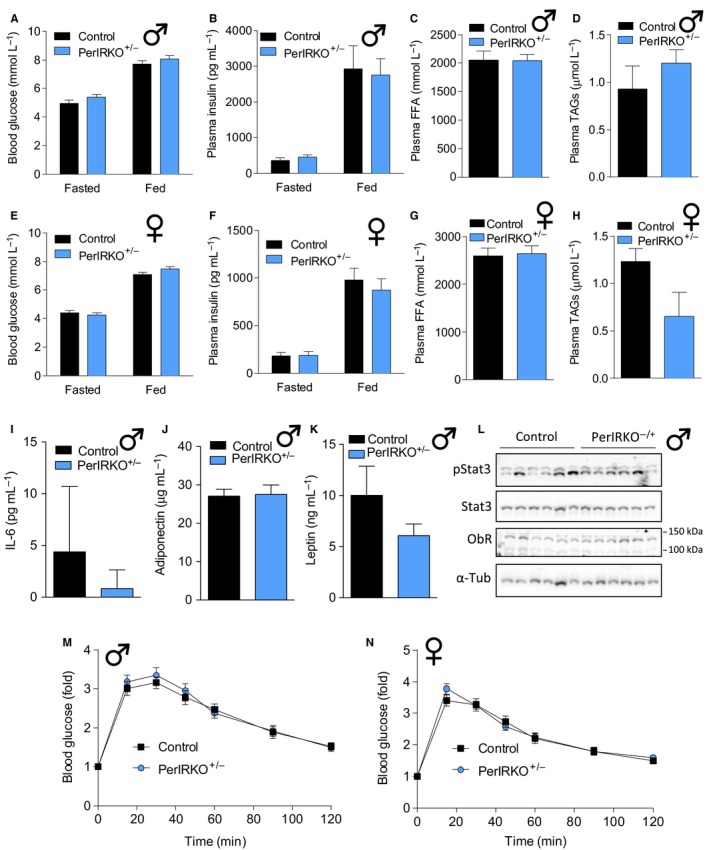
Partial peripheral tissue IR disruption does not affect glucose tolerance in adult mice. Four to six weeks following tamoxifen (TX) treatment, blood was collected from fed or 6‐h‐fasted male (A–D) and female (E–H) control and PerIRKO ^−/+^ mice and analysed for blood glucose or plasma insulin, free fatty acids (FFA) or triglycerides (TAGs). In male mice, plasma IL‐6, adiponectin and leptin levels were determined (I–K), and livers were immunoblotted for phosphorylation and total signal transducer and activator of transcription 3 (STAT3), leptin receptor (ObR) and α‐tubulin (α‐tub) (L). Glucose tolerance (GTT; 2 mg g^−1^ body weight) was determined in 5‐h‐fasted male (M) and female (N) control and PerIRKO ^−/+^ mice. Results are shown as means ± SE for *n* = 10–13 per group for blood glucose and GTT,* n* = 6 for immunoblots and 8–10 per group for all other parameters.

As insulin acts via the IR to regulate glucose uptake in peripheral tissue (fat and skeletal muscle) and to inhibit glucose production in the liver (Saltiel & Kahn, 2001), we next determined whether partial reduction in peripheral tissue IR expression alters glucose homeostasis. No differences were observed between control and PerIRKO^−/+^ fed and fasted blood glucose and plasma insulin, fasted plasma free fatty acids (FFA) or triglycerides (TG) for both males (Fig. 2A–D) and females (Fig. 2E–H), or male plasma IL‐6, adiponectin and leptin (Fig. 2I–K). PerIRKO^−/−^ have previously been reported to have enhanced hepatic leptin signalling (Koch *et al*., 2008); however, we found similar levels of leptin receptor and Stat3 phosphorylation in the liver of control and PerIRKO^−/+^ mice (Fig. 2L). Both male (Fig. 2M, and S3A,B, Supporting information) and female (Fig. 2N, and S3C,D, Supporting information) PerIRKO^−/+^ mice also showed normal glucose tolerance and glucose‐stimulated insulin secretion, suggesting that even a 50% reduction in peripheral IR expression insulin signalling was sufficient to maintain normal glucose homeostasis in adult mice.

**Figure 3:**
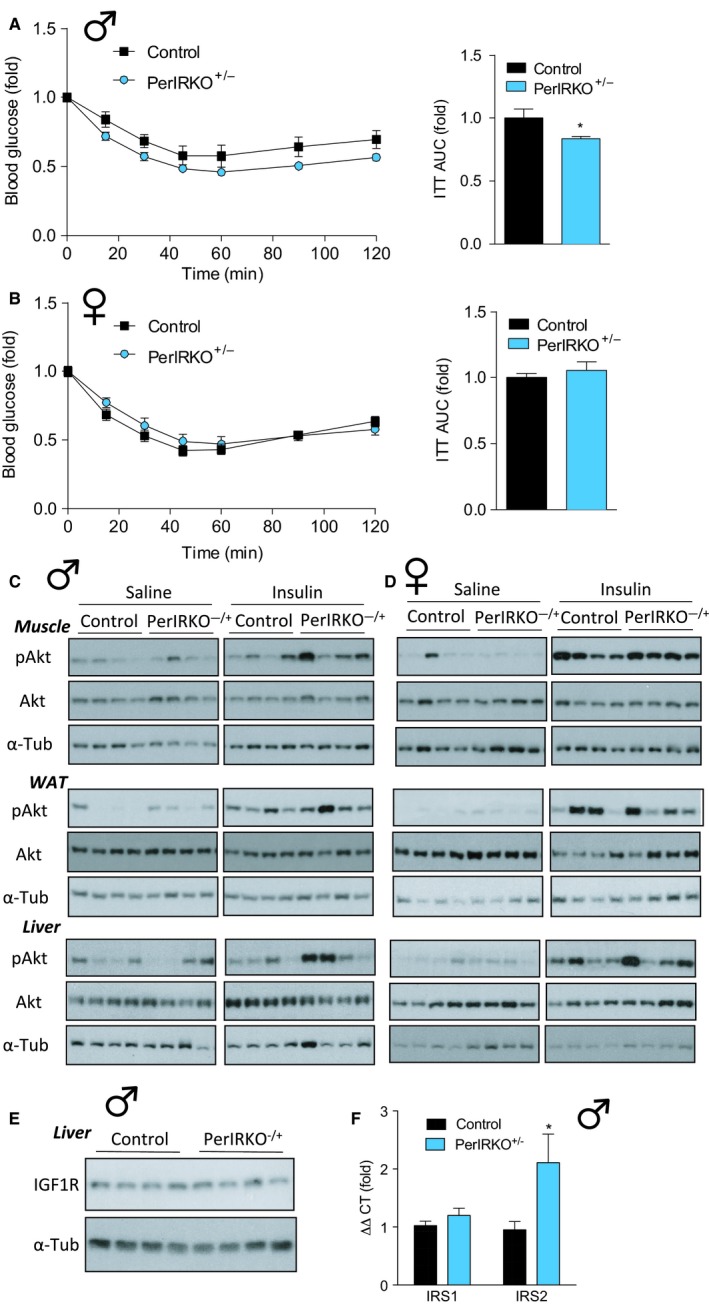
The effect of partial peripheral tissue IR disruption on insulin sensitivity of adult mice. Four to six weeks following tamoxifen (TX) treatment, insulin tolerance (ITT; 0.6 mU g^−1^ body weight) was determined in 2‐h‐fasted male (A) and female (B) control and PerIRKO ^−/+^ mice, and area under the curve (AUC) was calculated. Male (C) and female (D) control and PerIRKO ^−/+^ mice were fasted for 4 h and injected (intraperitoneal) with saline or insulin (0.6 mU g^−1^), and *gastrocnemius* muscle, white adipose tissue (WAT) and liver were extracted and processed for immunoblot analysis monitoring of Ser‐473 phosphorylated Akt (p‐Akt) and total Akt and α‐tubulin (α‐Tub). Insulin‐like growth factor 1 receptor (IGF1R) protein expression (E) and insulin receptor substrate (IRS) 1 and 2 mRNA levels (F) were determined in the livers of male mice. Results are shown as means ± SE for *n* = 6–11 per group. Significance was determined using two‐tailed Student's *t*‐test vs. control; *P < 0.05. A.U, arbitrary units.

To directly determine the effect of reduced peripheral tissue IR expression on insulin sensitivity, we performed insulin tolerance tests (ITT) and monitored for potential changes in insulin signalling in skeletal muscle, liver and white adipose tissue (WAT). Male PerIRKO^−/+^ mice showed a mild increase in relative whole‐body insulin sensitivity (as assess by ITT) (Fig. 3A), but no difference in absolute insulin sensitivity (Fig. S4A, Supporting information). Neither relative nor absolute insulin sensitivity was altered in female PerIRKO^−/+^ mice (Figs 3B and S4B, Supporting information).

The IR main target is the PI3K/Akt pathway which mediates the majority of insulin's metabolic effects (Saltiel & Kahn, 2001). We next assessed the ability of insulin to induce Akt Ser‐473 phosphorylation in peripheral tissue of control and PerIRKO^−/+^ mice to monitor for PI3K/Akt signalling. Following a 4‐h‐fast, mice were injected intraperitoneally with saline or a 0.6 mU g^−1^ insulin bolus, and tissues were extracted 10 min later. Basal (saline) or insulin‐stimulated Akt phosphorylation was not altered in *gastrocnemius* muscle or white adipose tissue (WAT) of male PerIRKO^−/+^ (Fig. 3C, and S2C,D, Supporting information), while the liver of male PerIRKO^−/+^s showed greater Akt phosphorylation than control in response to insulin (Fig. 3C, and S4E, Supporting information). This is consistent with the mild increase in relative whole‐body insulin sensitivity seen in male PerIRKO^−/+^ (Fig. 3A) and suggests that the ability of insulin to suppress hepatic glucose production is enhanced by partially reducing liver IR expression. Reduced IR expression did not affect basal or insulin‐induced Akt phosphorylation in the *gastrocnemius* muscle, WAT or liver of female PerIRKO^−/+^ mice (Fig. 3D, and S4F–H, Supporting information). We next determined whether there was a compensatory increase in signalling intermediates upstream of Akt in the livers of PerIRKO^−/+^ that may account for the increase in insulin‐stimulated hepatic Akt phosphorylation. While livers from control and PerIRKO^−/+^ showed similar insulin‐like growth factor 1 receptor (IGF1R) protein expression and insulin receptor substrate 1 (IRS) mRNA levels, PerIRKO^−/+^ had greater levels of IRS2 mRNA (Fig. 3E,F).

### Disruption of peripheral tissue IR expression does not extent lifespan of mice

**Figure 4:**
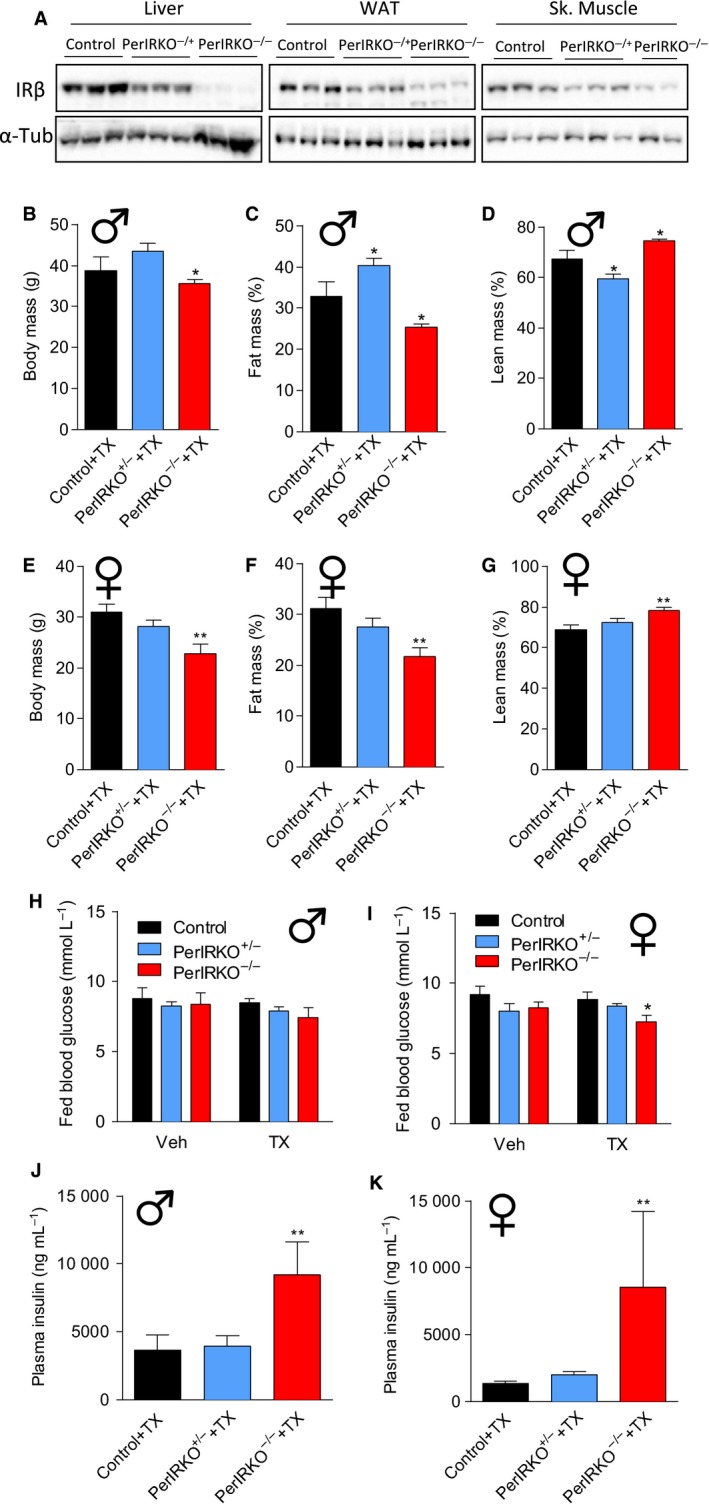
The effect of peripheral tissue IR disruption on body composition and blood glucose of aged mice. Control, PerIRKO ^−/+^ and PerIRKO ^−/−^ mice were aged for 35 weeks following tamoxifen (TX) treatment before insulin receptor (IRβ) expression was determined in the liver, white adipose tissue (WAT) or skeletal (Sk.) muscle (A). Male (B–D) and female (E–G) body mass and body composition (NMRI) were measured. Fed blood glucose and plasma inulin were determined in male (H, J) and female mice (I, K) 65 weeks following vehicle (Veh) or TX treatment. Results are shown as means ± SE for *n* = 4–10 per group for body composition, and *n* = 7–14 per group for blood glucose and plasma insulin. Significance was determined using one‐way ANOVA with LSD *post hoc* analysis; *P < 0.05, **P < 0.01.

Reduced IR expression in lower organisms (Friedman & Johnson, 1988; Kenyon *et al*., 1993; Morris *et al*., 1996; Clancy *et al*., 2001; Tatar *et al*., 2001), and reducing expression of aspects on the insulin signalling cascade (Holzenberger *et al*., 2003; Taguchi *et al*., 2007; Selman *et al*., 2008; Nojima *et al*., 2013) or fat‐specific ablation of the IR in mice extends lifespan (Blüher *et al*., 2003). Therefore, we determined the effect of partial (PerIRKO^−/+^) or complete (PerIRKO^−/−^) adult‐induced IR disruption in non‐neuronal tissues on aging. Importantly, we showed that IR expression of liver, white adipose tissue (WAT) and skeletal muscle was still substantially downregulated in aged PerIRKO^−/+^ and PerIRKO^−/−^ mice (Fig. 4A). At 50 weeks of age, both male and female PerIRKO^−/−^ mice had reduced body mass and relative fat mass, and increased relative lean mass (Fig. 4B–G). PerIRKO^−/+^ males showed increased relative fat mass and reduced relative lean mass, whereas female PerIRKO^−/+^ body composition was not different from control mice (Fig. 4B–G). In contrast to young PerIRKO^−/−^ mice, which had elevated fed blood glucose, at 80 weeks of age male PerIRKO^−/−^ mice had similar fed blood glucose, and female PerIRKO^−/−^ mice even had reduced blood glucose compared to control (Fig. 4H,I). However, fed plasma insulin levels in both male and female PerIRKO^−/−^ mice remained elevated at 80 weeks (Fig. 4J,K). Neither fed blood glucose or plasma insulin levels of male or female PerIRKO^−/+^ mice were different from that of control at 80 weeks of age (Fig. 4H–K).

**Figure 5:**
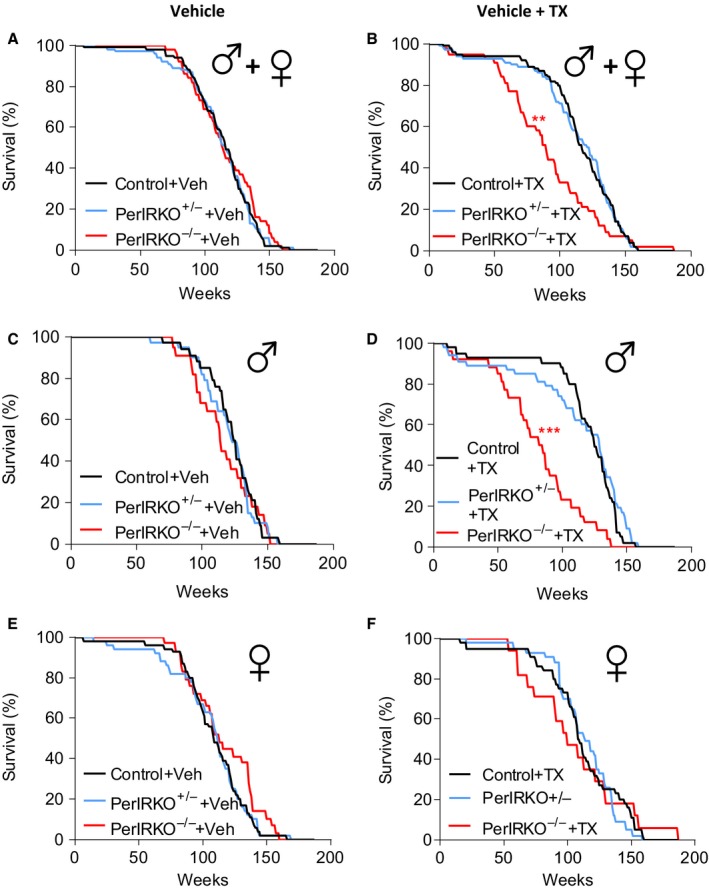
Peripheral tissue IR disruption does not affect lifespan of mice. Survival curves of combine male and female (A) vehicle (Veh)‐treated or (B) tamoxifen (TX)‐treated control, PerIRKO ^−/+^ and PerIRKO ^−/−^ mice. Survival curves for separated (C) Veh‐ or (D) TX‐treated males (*n* = 22–39 per group), and (E) Veh‐ or (F) TX‐treated females (*n* = 17–54 per group). Significance was determined using COX and log‐ranked regression analysis; **P < 0.01, ***P < 0.001 vs. control of the same treatment.

Next, we assessed the effect of partial or complete peripheral IR disruption on lifespan. We observed that TX‐treated PerIRKO^−/−^ mice had a reduced lifespan compared with control (Fig. 5B), an effect that was not observed in Veh‐treated control mice (Fig. 5A), indicating that shortened lifespan in PerIRKO^−/−^ was the result of peripheral IR disruption rather than floxP insertion into the IR gene. COX regression analysis indicated an interaction between genotype and gender (*P* = 0.03), and when separately analysed using log‐ranked regression, we found that the effect of complete peripheral tissue IR disruption on reducing lifespan was only observed in males (Fig. 5D,F). Partial reduction in IR expression in peripheral tissues (PerIRKO^−/+^) did not alter the lifespan of male or female mice (Fig. 5D,F).

**Figure 6:**
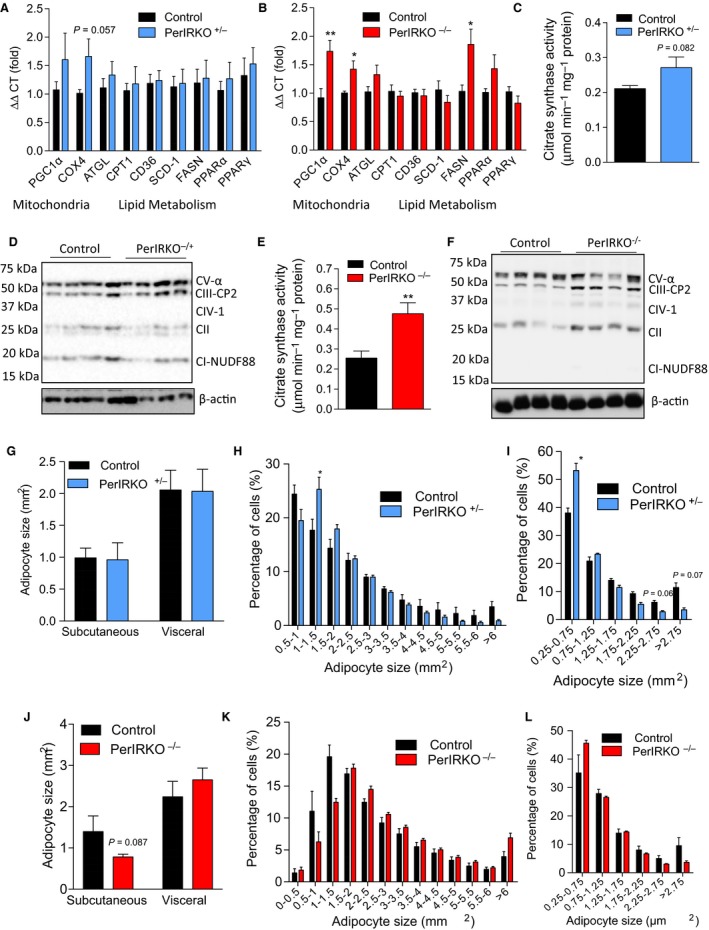
The effect of peripheral tissue IR disruption on adipocyte lipid metabolism and mitochondrial biogenesis. Markers of lipid metabolism (A–B), mitochondrial biogenesis (A–F) and adipocytes size (G–L) were examined in white adipose tissue from male PerIRKO ^−/−^ and PerIRKO ^−/+^ mice 4–6 weeks following tamoxifen (TX) treatment. Blots D and F are of mitochondrial electron transport chain components. Results are shown as means ± SE for *n* = 8–13 per group for mitochondrial and qPCR analysis and 4–7 for adipocyte size. *P < 0.05, **P < 0.01.

Adipose tissue insulin receptor knockout mice have been previously reported to have extended life expectancy (Blüher *et al*., 2003). In an effort to determine why our PerIRKO mice do not show a similar extension of lifespan, we sort to establish whether they have a similar white adipose phenotype of increased mitochondrial biogenesis and altered adipocyte size (Blüher *et al*., 2002; Katic *et al*., 2007). PerIRKO^−/−^, but not PerIRKO^−/+^, showed increased mitochondrial gene (PGC1α, COX4), protein (electron transport chain proteins) and enzyme activity (citrate synthase), indicating increased mitochondrial volume (Fig. 6A–F). While FASN was upregulated in PerIRKO^−/−^, no other lipogenic of lipolysis genes were upregulated in PerIRKOs (Fig. 6A,B). Despite young male PerIRKO^−/+^ mice having less total fat mass than controls (Fig. 1B,G), they had similar mean adipocyte size whereas PerIRKO^−/−^ tended to have reduced subcutaneous fat pad adipocyte size (Fig. 6G,H,J,K). In the visceral fat pads, both PerIRKO^−/+^ and PerIRKO^−/−^ mice tended to have a greater proportion of smaller adipocytes, and less larger adipocytes (Fig. 6I,L).

## Discussion

In the current study, we show that partial disruption of the IR in peripheral tissues of adult mice unexpectedly does not extended murine lifespan, while complete disruption reduces lifespan. This is despite partial IR disruption, surprisingly, slightly improving relative insulin sensitivity and reducing fat mass of young male mice, and complete disruption of the peripheral IR reducing fat mass in aged (50–80 weeks) mice.

In murine models, global ablation of the IR or IGF1R leads to early death (Liu *et al*., 1993; Accili *et al*., 1996; Holzenberger *et al*., 2003); however, partial disruption of the insulin/IGF1 signalling cascade has lifespan‐extending effects (Blüher *et al*., 2003; Holzenberger *et al*., 2003; Taguchi *et al*., 2007; Selman *et al*., 2008; Nojima *et al*., 2013). This suggests that in mammals, the extent of disruption of insulin/IGF1 signalling cascade, and specific tissue targeted, determines whether and which affect the intervention may have on lifespan. Our results extend these findings by showing that disrupting the IR in all peripheral tissues of adult mice to alter insulin/IGF1 signalling does not extend lifespan in mice. This is particularly surprising as an almost identical *C. elegans* model, that is a RNAi‐mediated non‐neuronal disruption of *daf‐2* signalling in adult worms, does show extended lifespan (Dillin *et al*., 2002).

One of the major functions of the IR is to maintain glucose homeostasis (Saltiel & Kahn, 2001). Consistent with this, and the findings of others (Accili *et al*., 1996; Kulkarni *et al*., 1999; Michael *et al*., 2000; Koch *et al*., 2008), we reported that complete disruption of the IR in peripheral tissue (PerIRKO^−/−^) resulted in postprandial glucose intolerance. Chronically elevated blood glucose levels resulting from impaired insulin sensitivity are associated with the pathogenesis of various diseases (DeFronzo & Ferrannini, 1991; Calle & Kaaks, 2004), and therefore are likely to be a key contributor to the reduced lifespan seen in mice with complete peripheral tissue IR disruption. Interestingly, the elevated fed blood glucose, which was observed in young PerIRKO^−/−^ mice, was normalized during aging. While this may indicate that during aging, the Cre‐recombinase activity reduced, fed plasma insulin levels remained elevated in aged PerIRKO^−/−^ mice, suggesting that either adaptions occurred to allow some glucose utilization independent of the IR or endogenous glucose production via hepatic gluconeogenesis was impaired in aged PerIRKO^−/−^. Along these lines, we also unexpectedly report that young PerIRKO^−/+^ mice have mildly improved whole‐body relative insulin sensitivity, and this is likely the result of enhanced hepatic insulin signalling promoting the suppression of gluconeogenesis. This may be related to the reduced visceral fat mass of male PerIRKO^−/+^ promoting increased levels of the insulin‐sensitizing hormone adiponectin to enhance hepatic insulin sensitivity (Barzilai *et al*., 1998, 1999), and/or the increased expression of the insulin signalling intermediate IRS2 to compensate for the 50% reduction in IR expression. While the purpose of the current investigation was to determine the effect of adult‐induced IR disruption expression on lifespan, the potential for reduced IR expression to promote the suppression of hepatic glucose production warrants further investigation particularly given that the inability to suppress gluconeogenesis is a key factor contributing to hyperglycaemia in type 2 diabetes (Lin & Accili, 2011).

Consistent with aged PerIRKO^−/−^ mice having lower relative fat mass, the IR has been shown to mediate fat storage in prenatal (Blüher *et al*., 2002, 2003) and adult‐induced (Koch *et al*., 2008) IR knockout mice. High fat mass correlates with reduced lifespan (Blüher *et al*., 2003; Muzumdar *et al*., 2008), but an excess accumulation of fat is almost always associated with insulin resistance and impaired glucose tolerance making it difficult to determine which has the greatest influence on life expectancy. Herein, we report that despite a reduction in fat mass in aged PerIRKO^−/−^ mice, the males had reduced lifespan. This may suggest that the extent of insulin resistance, particular in nonfat tissue, may be a more relevant determinant in life expectancy then fat mass itself, and is consistent with metformin enhancing insulin sensitivity and extending murine lifespan independent of body or fat mass (Martin‐Montalvo *et al*., 2013). Why complete peripheral IR disruption preferentially reduced the lifespan of male mice is unclear, but is supported by previous reports of gender‐specific effects of metabolic interventions on murine lifespan (Holzenberger *et al*., 2003; Bokov *et al*., 2011) and suggests that males are more susceptible to tissue damage and diseases associated with insulin resistance and impaired glucose homeostasis.

Partial disruption of the IGF1R has been reported to substantially extend the lifespan of male and female mice by some (Holzenberger *et al*., 2003), but only mildly extend the lifespan of male, but not female, mice by others (Bokov *et al*., 2011). We report that partial adult‐induced peripheral IR disruption did not affect lifespan. In the current study, we disrupted the IR in adulthood, while IGF1R partial disruption (heterozygous) or disruption of downstream insulin signalling intermediates has exclusively been induced in prenatally or in early development, resulted in a reduced body size, which was evident from early development, and maintained throughout life (Holzenberger *et al*., 2003; Selman *et al*., 2008; Nojima *et al*., 2013). Reduced body size is a common phenotype seen in murine models that have extended lifespans (Coschigano *et al*., 2000; Flurkey *et al*., 2001; Nojima *et al*., 2013), and appears to be the result of the downregulation of IGF1 signalling (Coschigano *et al*., 2000, 2003; Holzenberger *et al*., 2003). Given that aged PerIRKO^−/+^ mouse body mass was not different from that of control, and PerIRKO^−/−^ reduction in body mass did not occur late in life, this would suggest that targeting of the IR in peripheral tissue of adult mice does not substantially alter IGF1 effects on growth. While calorie restriction extends the lifespan of rodents when indicated in adulthood (Weindruch & Walford, 1982) and the downregulation of the insulin/IGF1 signalling cascade following development extends the lifespan of *C. elegans* (Dillin *et al*., 2002), it is possible that in mammals, a critical window during development exists where the specific downregulation of the IR/IGF1R must occur to promote adaptations that promote longevity.

Extension of murine lifespan through impairment of the insulin/IGF1 signalling is generally associated with mild impairments in glucose homeostasis (Coschigano *et al*., 2000, 2003; Holzenberger *et al*., 2003; Taguchi *et al*., 2007; Selman *et al*., 2008). Partial disruption of the IR in this study did not effect or slightly enhanced (males) insulin sensitivity and glucose tolerance, potentially suggesting that the level of disruption of the IR was insufficient to significantly downregulate insulin signalling and therefore is unlikely to have induced alterations in IGF1 levels, mitochondrial respiration or adaptations which have been previously associated with lifespan extension in insulin/IGF1 impairment models (Lithgow *et al*., 1995; Schulz *et al*., 2007; Zarse *et al*., 2012). Another possible reason that we did not observe an extension of lifespan in our model is that the IR was ablated specifically in peripheral tissue. Several studies have shown reducing IRS expression in the brain extends murine lifespan (Taguchi *et al*., 2007), whereas the majority of other genetic models which downregulate insulin/IGF1 are also likely have reduced central insulin/IGF1 signalling (Holzenberger *et al*., 2003; Selman *et al*., 2008; Nojima *et al*., 2013). Indeed, as Blüher *et al*. (2003) reported that adipose tissue‐specific deletion (FIRKO) of the IR extends the lifespan of mice, the aP2‐Cre models they used have been shown to target the central nervous system (Martens *et al*., 2010) and the phenotype of adipose tissue‐specific IR knockout achieved using the Adipoq‐Cre differs substantially from the aP2‐Cre model (Softic *et al*., 2016). Furthermore, our PerIRKO^−/−^ mice show many similar adipocyte characteristics (increased mitochondrial volume and altered adipocyte size) to those of the FIRKO (Katic *et al*., 2007) without an extension of lifespan. This may indicate that central insulin/IGF1 signalling plays a more prominent role in regulating longevity than peripheral.

Taken together, our data indicate that targeting insulin/IGF1 signalling by impairing IR expression in all peripheral tissues of adult mice is not a viable option to extend lifespan in mammals. This may suggest that (i) metazoal observations cannot be directly translated into mammals, (ii) the role of IGF1 signalling differs significantly from IR in regard to the regulation of murine lifespan, and (iii) IR signalling affects lifespan in a tissue‐specific and possibly neuronal‐specific manner.

## Experimental procedures

### Antibodies and reagents

Rabbit antibodies against phospho‐S473‐Akt, Akt, IGF1R, phospho‐Stat3 and Stat3 and mouse antibodies against α‐tubulin and insulin receptor‐β were purchased from Cell Signaling Technology (Beverly, MA, USA). Oxidative phosphorylation (mitochondrial electron transport chain) antibody was from Abcam (Cambridge, UK) and leptin receptor from Santa Cruz Biotechnology (TX, USA). Unless stated otherwise, all other reagents were purchased from Sigma‐Aldrich Chemicals (St. Louis, MO, USA).

### Murine breeding and housing conditions

Mice were maintained in a temperature‐controlled special pathogen‐free (SPF) facility with 12‐h light–dark cycle and *ad libitum* access to a soya protein‐free chow diet (S8022‐S005; ssniff Diets, Soest, Germany) and water. Peripheral tissue inducible insulin receptor (IR) knockout mice have been described previously (Koch *et al*., 2008) and the corresponding lines IR^lox/+^ (B6.129S4(FVB)‐*Insr* ^*tm1Khn*^/J) and CreER (B6.129‐*Gt(ROSA)26Sor* ^*tm1(cre/ERT2)Tyj*^/J) were used. Mice had been backcrossed on C57Bl/6N background and were bred in‐house by intercrossing IR^lox/+^ and inducible CreER mice to produce IR^lox/+;CreER/+^ and IR^lox/lox;CreER/+^, which are referred to as PerIRKO^**−/+**^ and PerIRKO^**−/−**^, respectively, where Per stands for peripheral. IR^lox/+;+/+^ and IR^lox/lox;+/+^ littermates were used as control mice. To induce Cre expression, mice were administered 2 mg tamoxifen (Cayman Chemicals, Ann Arbor, MI, USA) or carrier via oral gavage for five consecutive days. All experiments were approved by the respective Ethics Committees of the State of Ministry of Environment, Health and Consumer Protection (Federal States of Brandenburg and Thuringia, Germany), and the Canton of Zurich Veterinary Office, Switzerland.

### Metabolic, body composition and mortality measures

Fed and fasted blood was collected via submandibular bleeding, and blood glucose was determined using a hand‐held glucose meter (Bayer Contour XT Meter). Plasma insulin, IL‐6, leptin and adiponectin levels were determined using an immunoassay (Meso Scale Discovery, Gaithersburg, MD, USA) or by ELISA for rat insulin using a mouse insulin standard (Crystal Chem Inc., Chicago, IL, USA), and plasma free fatty acids (FFA) and triglycerides (TGs) by enzymatic reaction (Cobas Mira; La Roche, Basel, Switzerland). Insulin (ITT) and glucose (GTT) tolerance tests were performed in 2‐ and 5‐h‐fasted mice, respectively, by intraperitoneally injecting a bolus of insulin (0.6 mU g^−1^; ITT) or d‐glucose (2 mg g^−1^; GTT), and tail blood glucose was measured at the time points indicated as described previously (Merry *et al*., 2014). Citrate synthase activity was measured in supernatant by examining the increase of 5,5‐dithiobis‐2‐nitrobenzoate (DTNB) at a wavelength of 412 nm (Srere, 1969).

PhenoMaster (TSE Systems, Bad Homburg, Germany) open‐circuit calorimetry system was used to measure oxygen consumption, energy expenditure and respiratory exchange ratios (RER; VCO_2_/VO_2_) over 48 h (two light–dark cycles) following a 15‐ to 24‐h acclimation period. Body composition was measured by nuclear magnetic resonance (Echo MRI‐100 Body Composition Analyzer; Echo Medical Systems, Huston, TX, USA) as described previously (Weimer *et al*., 2014). For the aging study, mice were inspected daily for health concerns, and deaths were recorded, while moribund animals were sacrificed by a veterinarian scientifically not involved in the study, and recorded as dead.

### Immunoblotting

Immunoblotting was performed essentially as described previously (Merry *et al*., 2014). Briefly, snap‐frozen mouse tissue samples were homogenized using an electrical hand‐held homogenizer in 10–20 volumes of ice‐cold RIPA lysis buffer (50 mm HEPES [pH 7.4], 1% (vol/vol) Triton X‐100, 1% (vol/vol) sodium deoxycholate, 0.1% (vol/vol) SDS, 150 mm NaCl, 10% (vol/vol) glycerol, 1.5 mm MgCl_2_, 1 mm EGTA, 50 mm sodium fluoride, protein inhibitor cocktail [Roche], 1 mm phenylmethylsulfonyl fluoride, 1 mm sodium vanadate), incubated for 20 min on ice and centrifuged at 20,000 *g* for 60 min at 4 °C. The supernatants were resolved by SDS‐PAGE and processed for immunoblotting by standard procedures.

### Real‐time polymerase chain reaction

RNA was extracted from frozen WAT samples using TRIzol reagent (Invitrogen, Carlsbad, CA, USA), and mRNA was reverse‐transcribed using the High Capacity cDNA Reverse Transcription Kit (Applied Biosystems, Foster City, CA, USA). Quantitative real‐time PCR was performed on a ViiA™ 7 Real‐Time PCR System (Applied Biosystems) using the SYBR Green Select Master Mix (Applied Biosystems). Reactions were performed in duplicate and relative quantification was achieved using the ΔΔ*C*_t_ method with 18S ribosomal RNA as an internal control. Primer sequences used are listed in Table S1.

### Adipocyte histological analysis

Adipose tissues was fixed in phosphate‐buffered 4% formaldehyde and embedded in paraffin blocks. 6‐μm paraffin sections were prepared, mounted on slides and stained with haematoxylin–eosin (H&E). Images were acquired with 10× objective on Axio Scope.A1 microscope (Oberkochen, Germany) equipped with a AxioCam MRc digital camera (Oberkochen, Germany). Measurement of adipocyte size (as area in mm^2^) was performed with a custom‐designed macro using Fiji‐ImageJ.

### Statistical analyses

All data were presented as mean ± SEM. Statistical significance was determined for all data except lifespan analysis using unpaired two‐tailed Student's *t*‐test and one‐way ANOVA with Fisher's least significant difference (LSD) post hoc analysis as indicated. The level of significance was set at *P* < 0.05. Mortality rates during the aging study were assessed using log‐ranked and COX regression tests to compare survival (spss version 20, IBM, Armonk, NY, USA).

## Funding

This study was supported by the research programme of the Jena Centre for Systems Biology of Aging (JenAge) funded by the German Ministry for Education and Research (Bundesministerium für Bildung und Forschung/BMBF 0315581), the German Research Association (Deutsche Forschungsgemeinschaft, DFG) Research Training Group 1715 Molecular Signatures of Adaptive Stress Responses, Jena, Germany, the Swiss National Science Foundation (Schweizerischer Nationalfonds, SNF 31003A_156031), the Novartis Foundation (Biomedical Research Fund; 14C149) and the National Institutes of Health (USA) (NIH R01 DK031036).

## Author's contributions

TLM contributed to the design and execution of experiments and interpretation of results. DK, BL and DP executed experiments and contributed to experimental design. AFHP and CRK were involved in the design of the study, interpretation of results and supply of animal lines and reagents. KZ and MR contributed to the study design and interpretation of results. TLM, CRK, MR and KZ co‐wrote the manuscript.

## Conflict of interest

None declared.

## Supporting information


**Fig. S1** Insulin receptor (IR) expression is reduced in peripheral tissue from PerIRKO^−/−^ and PerIRKO^−/+^ mice.
**Fig. S2** The effect of complete peripheral tissue IR disruption on body composition and blood glucose regulation in adult mice.
**Fig. S3** Partial peripheral tissue IR disruption does not affect glucose tolerance or insulin secretion of adult mice.
**Fig. S4** The effect of partial peripheral tissue IR disruption on insulin sensitivity of adult mice.

**Table S1** Mouse qPCR primer sequences.
