# Supplementary figures and images for: Impairment of insulin signalling in peripheral tissue fails to extend murine lifespan
Source: Aging Cell. 2017 May 22;16(4):761–72. doi: 10.1111/acel.12610 (PMC5506415; doi:10.1111/acel.12610)

Supplementary Figure 1

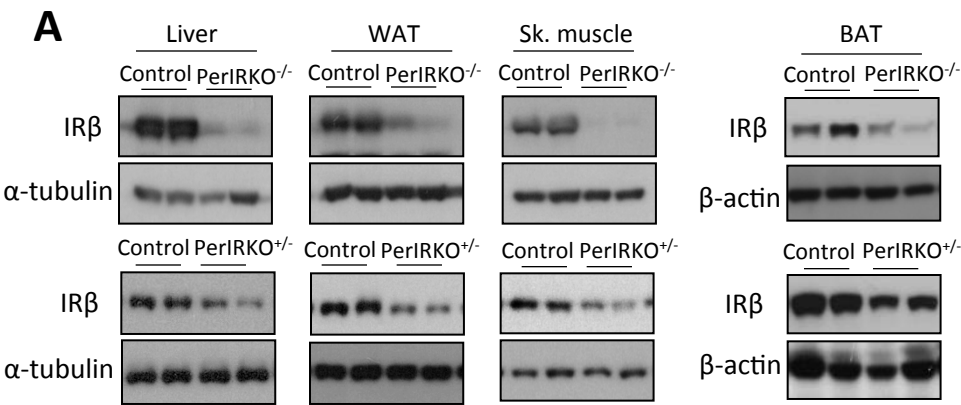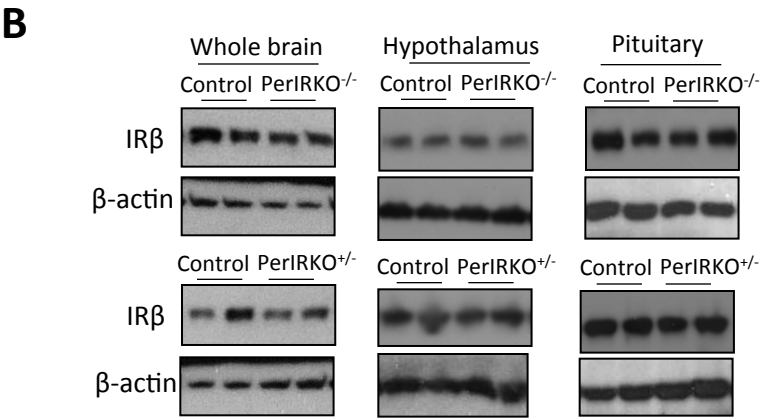

Supplementary Figure 2

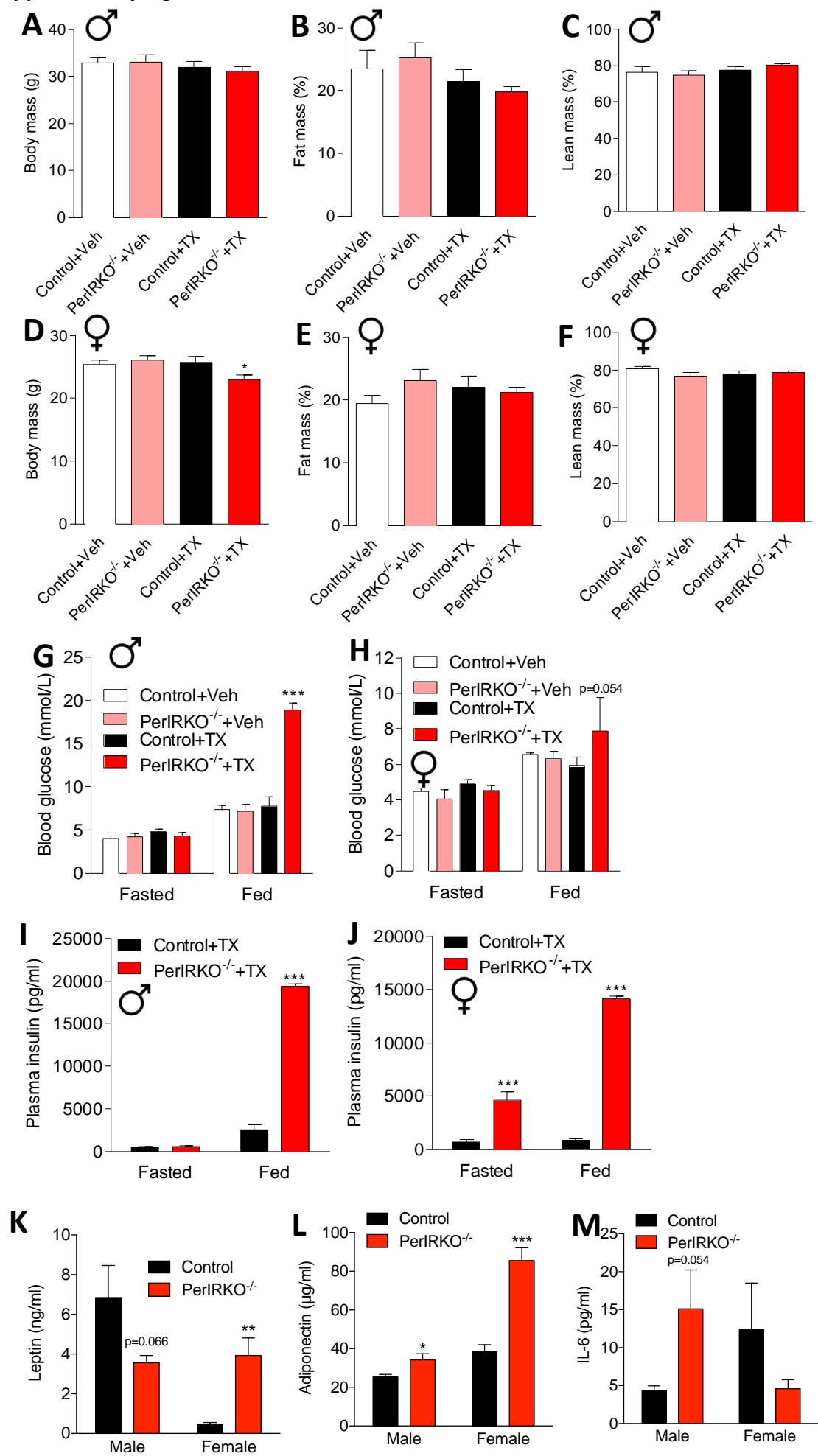

Supplementary Figure 3

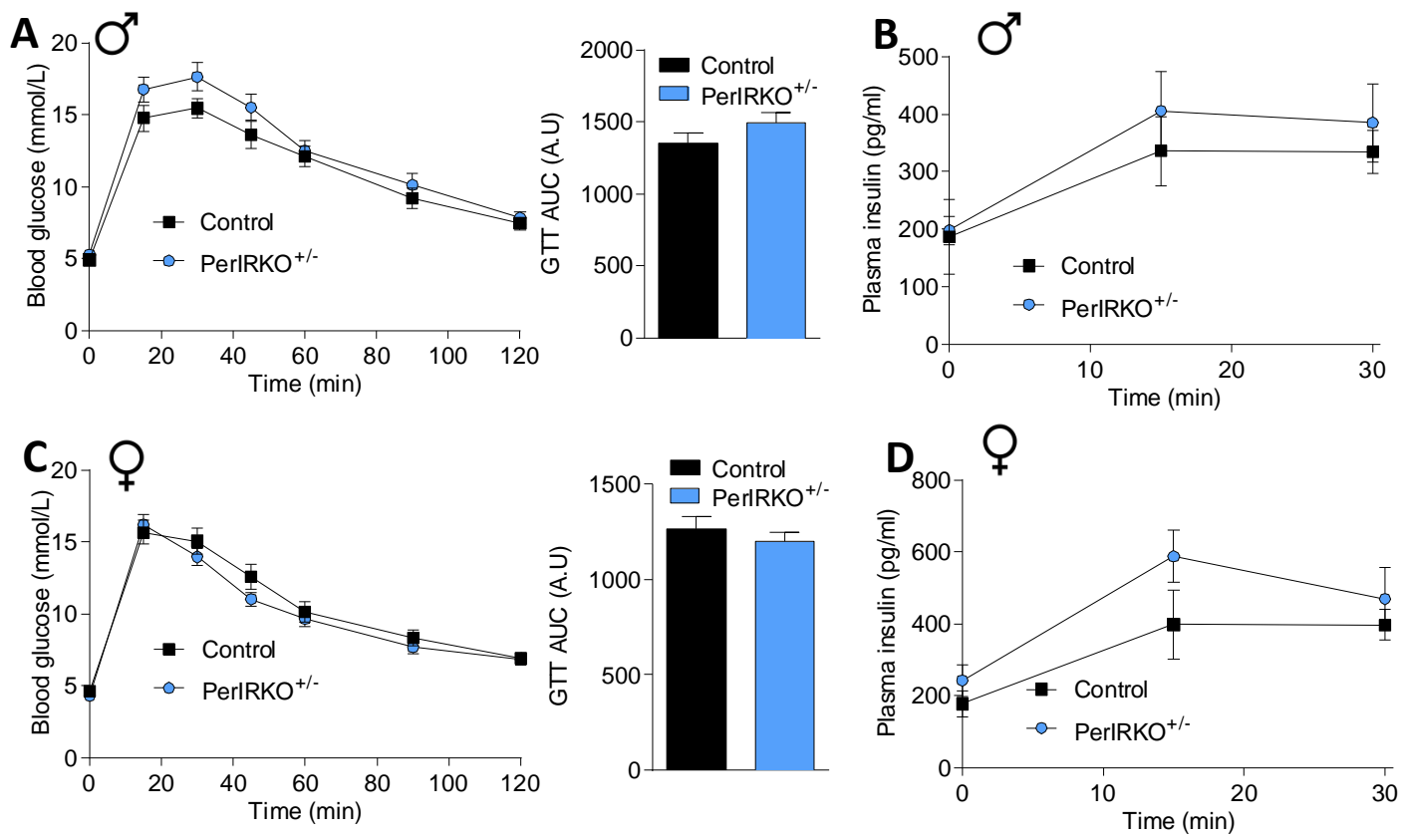

Supplementary Figure 4

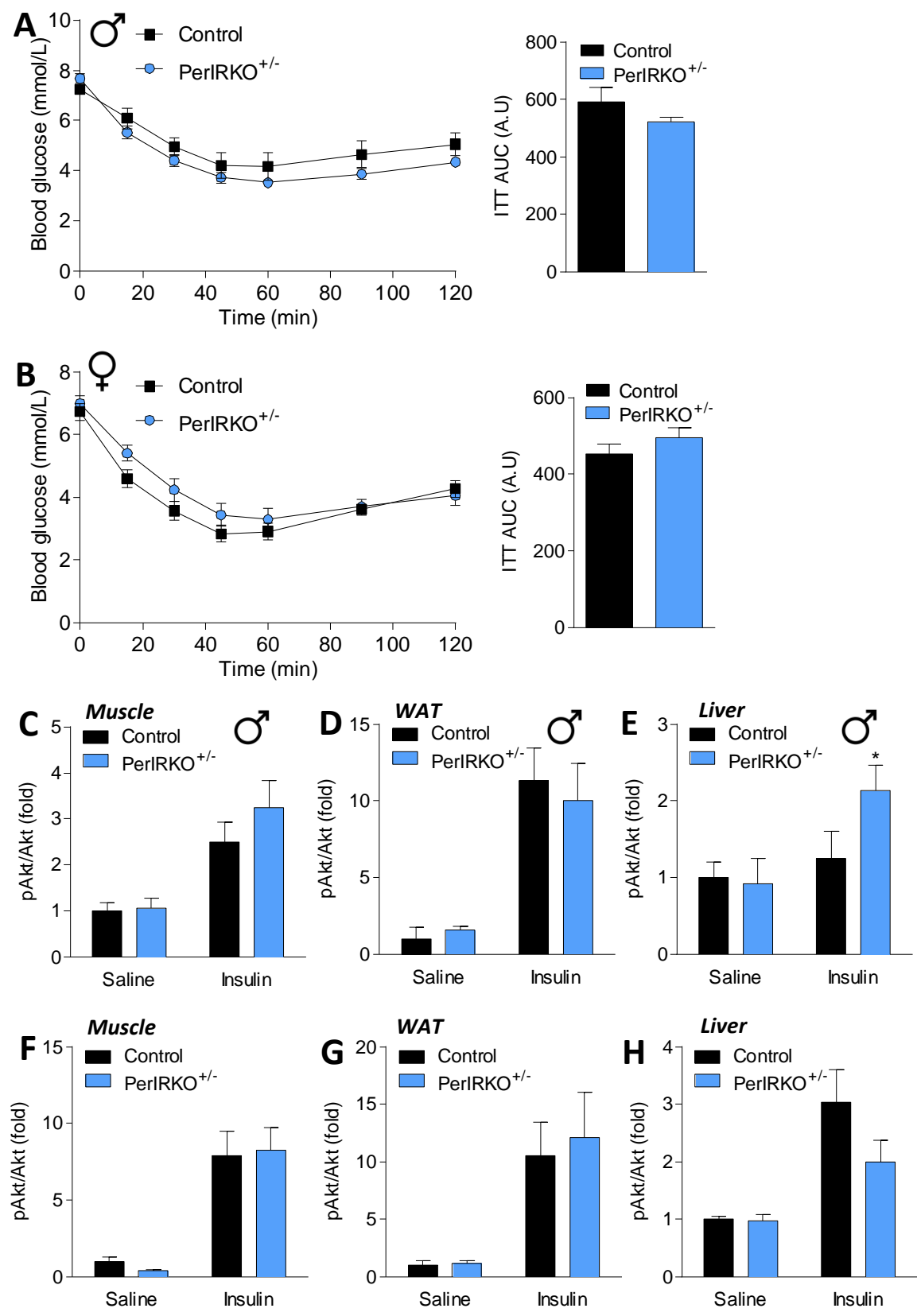

Supplement: Supplementary file 1 — Fig. S1 Insulin receptor (IR) expression is reduced in peripheral tissue from PerIRKO−/− and PerIRKO−/+ mice. Fig. S2 The effect of complete peripheral tissue IR disruption on body composition and blood glucose regulation in adult mice. Fig. S3 Partial peripheral tissue IR disruption does not affect glucose tolerance or insulin secretion of adult mice. Fig. S4 The effect of partial peripheral tissue IR disruption on insulin sensitivity of adult mice. [file ACEL-16-761-s001.pdf]
