# Supplementary material for: Impairment of insulin signalling in peripheral tissue fails to extend murine lifespan
Source: Aging Cell. 2017 May 22;16(4):761–72. doi: 10.1111/acel.12610 (PMC5506415; doi:10.1111/acel.12610)
Supplement: Supplementary file 2 — Table S1 Mouse qPCR primer sequences. [file ACEL-16-761-s002.docx]

**TABLES**

**Supplementary Table 1.** Mouse qPCR primer sequences.

| Gene name | Forward primer | Reverse primer |
| --- | --- | --- |
| 18S | GATCCATTGGAGGGCAAGTCT | CCAAGATCCAACTACGAGCTTTTT |
| COX4 | ACTACCCCTTGCCTGATGTG | GCCCACAACTGTCTTCCATT |
| PGC1α | AAGTGTGGAACTCTCTGGAACTG | GGGTTATCTTGGTTGGCTTTATG |
| ATGL | AACACCAGCATCCAGTTCAA | GGTTCAGTAGGCCATTCCTC |
| CPT-1 | GAACCCCAACATCCCCAAAC | TCCTGGCATTCTCCTGGAAT |
| CD36 | GCCAAGCTATTGCGACATGA | ATCTCAATGTCCGAGACTTTTCAAC |
| SCD-1 | TGGGTTGGCTGCTTGTG | GCGTGGGCAGGATGAAG |
| FASN | TTCCAAGACGAAAATGATGC | AATTGTGGGATCAGGAGAGC |
| PPARα | ACAAGGCCTCAGGGTACCA | GCCGAAAGAAGCCCTTACAG |
| PPARγ | CAAGAATACCAAAGTGCGATCAA | GAGCTGGGTCTTTTCAGAATAATA |
| IRS1 | GCGGGCTGACTCCAAGAAC | GCTATCCGCGGCAATGG |
| IRS2 | GGAGAACCCAGACCCTAAGCTACT | GATGCCTTTGAGGCCTTCAC |
